# Experiences of Transgender and Gender Diverse Patients in Accessing Gender-Affirming Chest Masculinization: A Systematic Review and Qualitative Thematic Synthesis of the Literature

**DOI:** 10.1177/22925503261477385

**Published:** 2026-08-26

**Authors:** Emma Lim, Justin Haas, Precious Adekoya, Sophia Hou, Kathleen Armstrong, Helene Retrouvey, Ammara Ghumman

**Affiliations:** 1 Department of Undergraduate Medicine, 3710 Michael G. DeGroote School of Medicine, McMaster University, Hamilton, ON, Canada; 2 Division of Plastic Surgery, Department of Surgery, 3710 McMaster University, Hamilton, ON, Canada; 3 School of Medicine, 4257 Queen's University, Kingston, ON, Canada; 4 Women's College Research Institute, 7938 Women's College Hospital, Toronto, ON, Canada; 5 Division of Plastic and Reconstructive Surgery, University of Toronto, Toronto, ON, Canada

**Keywords:** chest surgery, masculinizing chest surgery, non binary, gender-affirming care, top surgery, transgender, gender diverse, non binary, chirurgie thoracique, torsoplastie masculinisante, affirmation de genre, mastectomie masculinisante, transgenre, diversité de genre, non binaire

## Abstract

**Introduction:**

The objective of this review was to synthesize existing qualitative research regarding the experiences of transgender and gender diverse (TGD) individuals undergoing gender-affirming chest masculinization surgery, in order to identify critical barriers and facilitators which may affect access to and quality of care.

**Methods:**

We searched eight electronic databases in August 2025 for qualitative results analyzing the experiences of TGD individuals undergoing chest masculinization. We included primary qualitative or mixed-methods studies published in English/French that involved trans masculine or gender-diverse individuals ≥12 years undergoing chest masculinization. Exclusion criteria included cisgender or trans feminine populations, nonsurgical or nongender-affirming care, absence of qualitative data, and nonpeer-reviewed literature. The Critical Appraisal Skills Programme checklist was used to appraise the quality of included studies. A thematic synthesis was conducted following Thomas and Harden.

**Results:**

Sixty studies met the inclusion criteria. Four analytical themes were identified: (1) charting a course, (2) interactions with providers, (3) surgical goal setting, and (4) adjusting to oneself.

**Conclusion:**

TGD individuals face persistent structural and interpersonal barriers when accessing chest masculinization surgery. The domains identified in the present review form a patient experience-driven framework that healthcare stakeholders and providers can use to improve access to care. Healthcare providers should be aware that nonbinary and binary transgender patients may have varying surgical goals for chest masculinization.

## Résumé

**Introduction:**

La présente analyse visait à synthétiser les recherches qualitatives existantes sur les expériences des personnes transgenres et de diverses identités de genre (DIG) qui se soumettent à une torsoplastie masculinisante d’affirmation de genre, afin d’établir les obstacles et les leviers susceptibles d’influer sur l’accès aux soins et la qualité des soins.

**Méthodes:**

Les chercheurs ont fouillé huit bases de données électroniques en août 2025 afin d’en extraire des résultats qualitatifs sur les expériences de personnes DIG qui avait subi une torsoplastie masculinisante. Les critères d’inclusion étaient les personnes transmasculine ou de diverses identités de genre âgées de 12 ans et plus qui avaient subis une torsoplastie masculinisante, ainsi que les études primaires qualitatives ou à méthodologie mixte (en anglais et en français). Les critères d’exclusion englobaient les populations cisgenres ou transféminines, les soins non chirurgicaux ou non liés à l’affirmation de genre, l’absence de données qualitatives et les publications sans comité de lecture. Les chercheurs ont utilisé les listes du Critical Appraisal Skills Programme pour évaluer la qualité des études retenues, puis ont réalisé une synthèse thématique selon la méthode de Thomas et Harden.

**Résultats:**

Au total, 60 études respectaient les critères d’inclusion. Quatre thèmes analytiques ont été dégagés : 1) plan d’action, 2) interactions avec les prestataires de soins, 3) établissement des objectifs chirurgicaux, et 4) adaptation à soi.

**Conclusion:**

Les personnes DIG font face à des obstacles structurels et interpersonnels persistants en matière d’accès à la torsoplastie masculinisante. Les domaines relevés dans la présente analyse forment un cadre axé sur l’expérience du patient que les intervenants et les prestataires de soins peuvent utiliser pour améliorer l’accès aux soins. Les prestataires de soins devraient savoir que les objectifs de torsoplastie masculinisante des patients transgenres non binaires et binaires peuvent varier.

## Introduction

Chest masculinization surgery, colloquially referred to as “top” surgery, is a gender-affirming procedure that typically involves resection of glandular breast tissue. It is often the first or only surgical procedure that many transgender and gender-diverse (TGD) undergo^1,2^ and is the most commonly requested gender-affirming surgery requested, with demand increasing over recent years.^1-3^ Chest masculinization is associated with improved mental health outcomes, increased quality of life,^4-6^ and decreased gender dysphoria.^7,8^

Many TGD individuals face challenges accessing chest masculinization surgery. In a 2024 Canadian survey, all TGD patients undergoing gender-affirming surgery reported difficulty accessing care.^9^ Barriers to care are often systemic, encompassing challenges like social stigma, safety, cost, lack of insurance coverage, and geographical inaccessibility.^10-14^ Significantly, these barriers have been linked to social difficulties, adverse health outcomes, and increased healthcare consumption.^15-17^ In response, some people may seek private care despite financial strain or choose to avoid medical systems entirely.^4,18^

Understanding patient experiences is essential in improving the quality and accessibility of gender-affirming care. However, research into the experiences of patients navigating chest masculinization remains limited. The current body of transgender literature often groups binary and nonbinary experiences under one umbrella, despite evidence of greater health disparities in nonbinary populations.^18,19^ Additionally, nonbinary patients face greater misunderstanding from primary care physicians and may have distinct transition and surgical goals.^20,21^

Thus far, no prior reviews have explored how TGD individuals interact with healthcare systems while accessing chest-masculinization surgery. The objective of this review was to synthesize existing qualitative research regarding the experiences of undergoing gender-affirming chest masculinization surgery, in order to identify critical barriers which may affect access to and quality of care.

## Methods

### Design

We conducted a qualitative evidence synthesis following thematic synthesis methodology outlined by Thomas and Harden.^22^ This protocol was registered on the Prospective Register of Systematic Reviews (registration number: CRD420251113988) in line with the Cochrane guidelines and the Enhancing Transparency in Reporting the Synthesis of Qualitative Research framework.^23,24^

### Selection Criteria

We included primary studies with a qualitative component, including mixed-methods studies, that involved trans masculine or gender-diverse individuals aged ≥12 years seeking chest masculinization surgery and that were published in English or French. No date restrictions were applied. Exclusion criteria included cisgender or transfeminine populations, nongender-affirming or nonsurgical care, absence of qualitative data, nonprimary or nonpeer-reviewed literature (excluding grey literature), and non-English/French publications. Studies were also excluded if experiences of chest masculinization could not be separated from other forms of care.

### Search Strategy

EMBASE, MEDLINE, Web of Science, SCOPUS, PsycINFO, Social Sciences Citation Index, CINAHL, and EBSCOhost LGBTQ+ Source were searched in August 2025. The search terms were developed using the Population, Exposure, Outcomes framework (Supplementary Material).^25^ Given the previously described difficulties in locating qualitative studies,^22^ we screened the reference lists of all included studies for additional research. Gray literature except university doctoral theses was excluded due to lack of peer-review.

### Screening

All titles and abstracts were screened in duplicate using Covidence.^26^ Discrepancies were resolved through discussion. Full texts were screened by three independent reviewers.

### Quality Appraisal

The quality of included studies was assessed using the Critical Appraisal Skills Programme (CASP) checklist.^27^ Quality assessment was used solely to inform confidence in our findings; it was not used to exclude studies.^28,29^

### Data Analysis

Data were extracted by three reviewers and included study characteristics and participant demographics. Primary data (eg, quotations, survey responses, field notes) were entered verbatim into NVivo15 for qualitative analysis (QSR International, MA, USA).

Thematic synthesis was conducted by three independent reviewers following the three-step approach described by Thomas and Harden, which consists of three analytical stages: (1) line-by-line coding of primary study findings, (2) development of descriptive themes, and (3) generation of analytical themes.^22^ The themes and subthemes derived from the main themes were summarized as text, with the writing of this publication representing the final analytic stage.^30^ Themes were constructed as patterns of shared meaning, therefore the importance of a theme was not contingent on how frequently it occurred, but if it captured something of relevance to the research. Even themes which appeared infrequently across studies were considered analytically significant and considered for inclusion in our analysis.^30^

### Reflexivity

Our research team includes individuals with diverse gender identities, sexualities, and backgrounds, including genderqueer/nonbinary, cisgender women, and cisgender men. Multiple authors identify as part of the LGBTQ2+/queer community. We acknowledge that qualitative research is inherently subjective, and our findings are shaped by our varied personal experiences. As described by Braun and Clarke (2022), our findings are inherently interpreted through the lens of the authors’ lived experiences. A continuous reflexive process was used throughout, including reflexive journaling and team-based reflexive discussion.^31^

## Results

**Figure 1.**
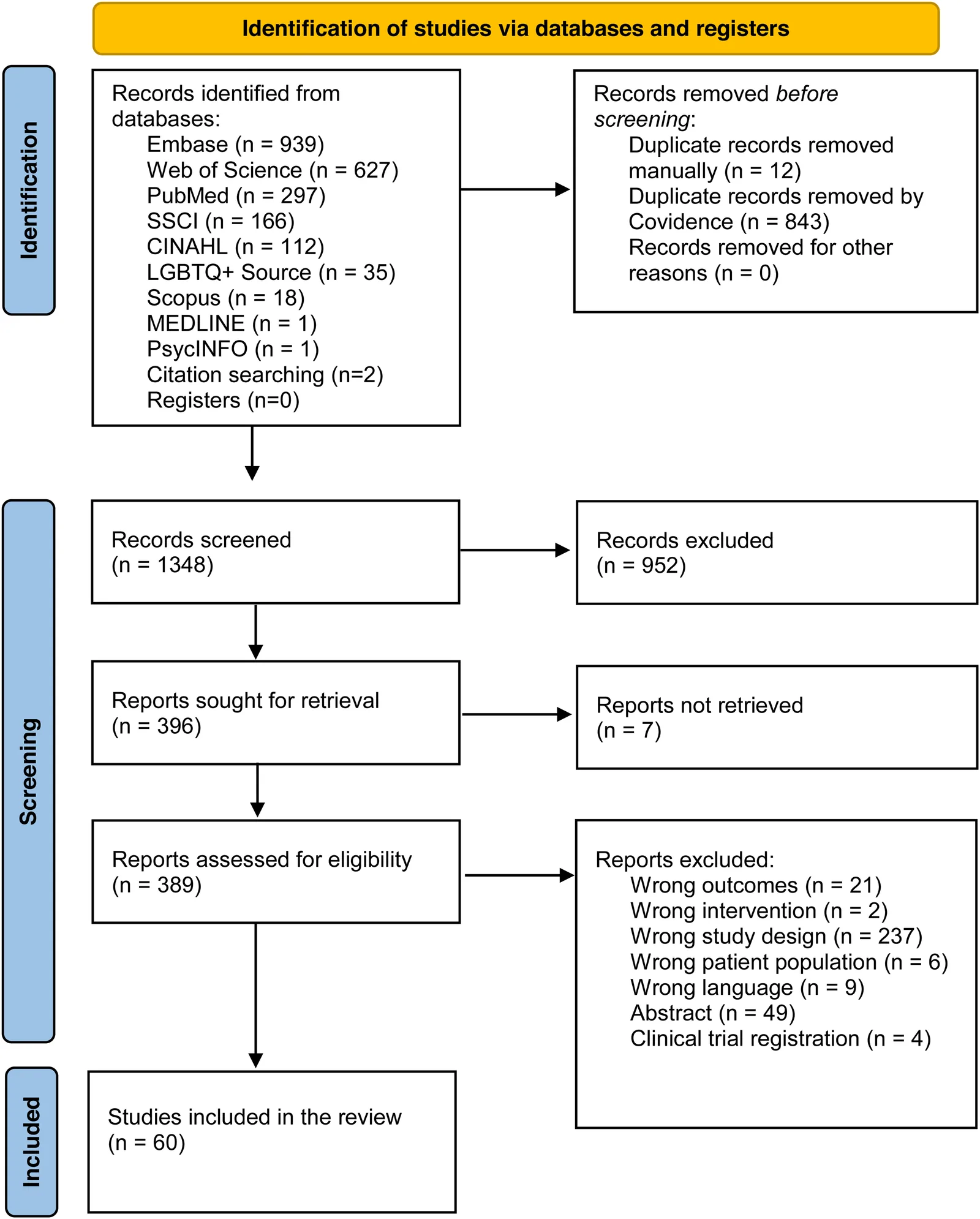
Preferred Reporting Items for Systematic reviews and Meta-Analyses^32^ Flow Diagram. SSCI, Social Science Citation Index; CINAHL, Cumulative Index to Nursing and Allied Health Literature.

A total of 2201 records were identified, after de-duplication 1348 reccords were screened, and 396 underwent full-text screening. Of these, 60 studies met the inclusion criteria and were included in our qualitative synthesis.^14,33-91^ Reasons for exclusion are detailed in the Preferred Reporting Items for Systematic Reviews and Meta-Analyses (PRISMA) flow diagram (Figure 1).^32^

### Study Characteristics

Of the 60 studies which met inclusion criteria, 35 were qualitative,^14,33-39,41,44-46,49-52,61-65,67,68,70,72,73,75-77,79,80,86-88,90^ and 25 were mixed methods.^40,42,43,47,48,53-60,66,69,71,74,78,81-85,89,91^ Included studies were published between 2009 and 2025 with study participants recruited primarily from North America^14,33,34,36,37,39-42,44-56,59-62,64,65,67,70,71,73,74,76-81,83-86,88^ (n = 44), Europe^36,43,44,60,62,69,70,76,88^ (n = 9), Oceania^58,60,62,90^ (Australia) (n = 4), Asia^33,73^ (n = 2), South America^59^ (n = 1), and Africa^67^ (n = 1). Four studies recruited participants from the internet^39,64,83,91^ (Table 1). The quality of included studies is presented below (Table 2).

**Table 1.** Characteristics of Included Studies.

Authors (Year)	Country	Sample	Age	Study Design	Analytic Strategy
Raner et al (2024)^14^	Canada	n = 108 Transgender man (n = 37) Nonbinary (n = 71)	25.54 ± 8.2 30.90 ± 9.6	Cross-sectional survey	
Agarwal and Dhillon (2023)^33^	India	n = 2	22 and 26	Semistructured interviews	Interpretive phenomenological analysis
Bauer et al (2009)^34^	Canada	n = 85	18+	Focus groups	Grounded theory
Bell and Purkey (2019)^35^	Canada	n = 11 Transgender man (n = 4) Transgender woman (n = 6) Gender diverse (n = 1)	46.5 (range 24-64)	Semistructured interviews	Phenomenological approach
Bjørnson and Sagbakken (2023)^36^	Norway	n = 10	NR	Interviews	Symbolic interactionism
Boyer et al (2023)^37^	USA	n = 30	NR	Semistructured interviews	Content analysis
Bridgwater et al (2024)^38^	Canada	n = 10 Transgender man (n = 1) Transgender woman (n = 2) Gender diverse (n = 7)	22.9 (range 18-34)	Focus groups and semistructured interviews	Qualitative description
Cavic et al (2025)^39^	Online	250 videos	NR	TikTok videos	Reflexive thematic analysis
Chang et al (2023)^40^	USA	n = 182 Male (n = 18) Female (n = 37) Transgender man (n = 35) Transgender woman (n = 51) Nonbinary (n = 28) Genderqueer (n = 2) Genderﬂuid (n = 1) Gender nonconforming (n = 2) Agender (n = 1) Other (n = 7)	33.8 ± 12.1	Survey	
Christiano et al (2024)^41^	USA	Transgender man (n = 53)	30.3 ± 12.2	Semistructured interviews	Content analysis
Crabtree et al (2024)^42^	USA	n = 150 Transgender man (n = 129) Transgender woman (n = 21)	18.6	Anonymous survey	Phenomenology
D'Angelo et al (2025)^43^	USA	n = 28 Male (n = 9) Transgender man (n = 18) Nonbinary (n = 1)	34.53 ± 8.11	Semistructured interviews	Reflexive thematic approach
de Brouwer et al (2021)^44^	EU	n = 257 Transgender man (n = 122) Transgender woman (n = 119) Nonbinary/other (n = 16)	NR	Survey	Phenomenology
del Mar Pastor Bravo and Linander (2024)^45^	Sweden, Spain	n = 34 Transgender man (n = 22) Transgender woman (n = 6) Nonbinary (n = 6)	15-26	Semistructured interviews	Abductive approach
Dozier (2023)^46^	USA	n = 30 Transgender man (n = 15) Male (n = 7) Nonbinary (n = 4) Other (n = 4)	31 (range 21-61)	Semistructured interviews	Inductive thematic analysis
Dupasquier et al (2025)^47^	Canada	n = 21 Transgender man (n = 9) Transgender woman (n = 7) Nonbinary/Agender/Genderqueer (n = 5)	38.7 ± 12.4 (range 24-59)	Focus groups	Social constructionism and reflexive thematic analysis
El-Hadi et al (2018)^48^	USA, Canada	n = 32 Transgender man (n = 12) Transgender woman (n = 20)	36 (range 18-81)	Survey	
Ferrin et al (2024)^49^	USA	n = 50 Transgender man (n = 20) Nonbinary/Genderqueer/Gender nonconforming (n = 24) Agender (n = 3) Other (n = 3)	NR	Survey	Inductive thematic analysis
Frohard-Dourlent et al (2020)^50^	Canada	n = 35 Transgender woman (n = 19) Transgender man (n = 13) Nonbinary (n = 8)	24-69	Interviews	Thematic analysis
Gillani et al (2025)^51^	USA	n = 35 Transgender man (n = 11) Transgender woman (n = 16) Nonbinary (n = 7) Other (n = 1)	18-25 (n = 4) 26-40 (n = 24) 41-60 (n = 7)	Focus groups	Reflexive thematic analysis
Goetz and Arcomano (2023)^52^	USA, Canada	n = 54 Transgender man/trans masculine (n = 17) Transgender woman/transfeminine (n = 6) Nonbinary (n = 9) Transgender nonbinary (n = 3) Nonbinary trans masculine (n = 8) Nonbinary trans feminine (n = 3) Nonbinary genderqueer (n = 5) Trans masculine genderqueer (n = 1) Nonbinary genderfluid (n = 2)	29 ± 9 (range 18-59)	Semistructured interviews	
Harry-Hernandez et al (2020)^53^	USA	n = 37 Transgender man (n = 12) Transgender woman (n = 11) Nonbinary/genderqueer (n = 14)	18-21 (n = 10) 22-25 (n = 5) 26-29 (n = 6) 30–33 (n = 9) 34–37 (n = 4) 38+ (n = 6)	Semistructured In-depth interviews	
Houghtaling (2023)^54^	USA	n = 8	16-20	Advisory group	
Hu et al (2023)^55^	USA	n = 67 Nonbinary (assigned male at birth) (n = 57) Nonbinary (assigned female at birth) (n = 10)	30.7 ± 11.3	Retrospective chart review	
Hu et al (2025)^56^	USA	n = 14 Assigned female at birth (n = 9) Assigned male at birth (n = 5)	32.5 (range 21-60)	Semistructured interviews	Interpretive description
Katz-Wise et al (2017)^57^	USA	n = 16 Transgender boy (n = 9) Transgender girl (n = 5) gender fluid (n = 1) Girlish boy (n = 1)	7-18	Semistructured interviews	Grounded theory
Kloer et al (2023)^58^	USA	n = 246 Transgender man (n = 56) Transgender woman(n = 80) Male (n = 17) Female (n = 31) gender nonconforming (n = 38) Genderqueer (n = 23) Agender (n = 1)	42	Survey and telephone interviews	Thematic analysis
Lee et al (2019)^59^	Australia	n = 10 Transgender man (n = 4) Nonbinary (n = 6)	18-36	Semistructured interviews	Exploratory inductive thematic analysis
Lerri et al (2025)^60^	Brazil	n = 20	26.5 (range 18−37)	Interviews	Content analysis
Littman (2021)^61^	USA, Canada, Australia, UK	n = 100 Assigned female at birth (n = 69) Assigned male at birth (n = 31)	29.2 ± 9.1	Survey	
MacCormick et al (2024)^62^	Canada	n = 21 Transgender man (n = 11) Transgender woman (n = 3) Nonbinary/Genderqueer (n = 7)	18-29 (n = 9) 30-39 (n = 9) ≥40 (n = 3)	Semistructured interviews	Qualitative description and phenomenology
MacDonald et al (2016)^63^	North America, EU, and Australia	Transgender man (n = 22)	24-50	Semistructured interviews	Qualitative and interpretive description methodology
Makhoul et al (2023)^64^	USA	n = 36	34 (range 25-51)	Semistructured interviews	Grounded theory approach
Martin and Coolhart (2022)^65^	Online	n = 10 Transgender man (n = 6) Transgender woman (n = 3) Nonbinary (n = 1)	39.7 ± 15.6 (range 24-69)	Semistructured interviews	Interpretive phenomenology
Mathis (2021)^66^	USA	n = 22 Male (n = 9) Female (n = 8) Transgender woman (n = 3) Intersex (n = 1) Nonbinary (n = 1)	19-72	Semistructured interviews	Transformative paradigm, discursive aggression, and queer of color critique
McTernan et al (2020)^67^	USA	n = 776 Transgender woman (n = 665) Nonbinary (n = 111)	32.1 ± 8.7 (range 15-61) 28.7 ± 9.8 (range 15-68)	Semistructured interviews	NR
Miller (2019)^68^	South Africa	n = 6 Transgender man (n = 4) Transgender woman (n = 2)	30 ± 3.69 (range 26-35)	Survey and retrospective chart review	Social constructionism
Mousavian et al (2024)^69^	USA	n = 15 Transgender man (n = 5) Transgender woman (n = 6) Nonbinary (n = 4)	NR	Semistructured interviews	Grounded theory
Nieder et al (2021)^70^	Germany	n = 75 Transgender man (n = 59) Transgender woman (n = 11) Nonbinary (n = 5)	12-21	Survey	Cross-sectional
Pipkin and Clarke (2024)^71^	UK	n = 6 Man (n = 2) Transgender man (n = 2) Nonbinary (n = 2)	23-41	Focus groups	Inductive thematic analysis
Poudrier et al (2019)^72^	USA	Transgender man (n = 58)	NR	Survey	Cross-sectional
Puckett et al (2018)^73^	USA	n = 221 Transgender man (n = 100) Transgender woman (n = 66) Nonbinary (n = 55)	28.4	Diaries/survey	Grounded theory
Raghuram (2024)^74^	India	n = 4 Transgender man (n = 1) Transgender woman (n = 2) Nonbinary (n = 1)	NR	Semistructured interviews	Exploratory case study
					
Roller et al (2015)^75^	USA	n = 25 Assigned male at birth (n = 5) Assigned female at birth (n = 20)	21-64	Semistructured interviews	Grounded theory
Ross et al (2024)^76^	Amsterdam	n = 21	38.1	Semistructured interviews and focus groups	Thematic analysis
Ross et al (2016)^77^	Canada	n = 10 Transgender woman (n = 4) Transgender man (n = 1) Friends/Family (n = 3) Healthcare provider (n = 2)	18-65	Semistructured interviews	Case study
Schafer et al (2024)^78^	USA	n = 302 Nonbinary (n = 38) Transgender man (n = 264)	28 ± 7 26 ± 8	Retrospective survey study
Slothouber (2024)^79^	Canada	n = 19 Assigned female at birth (n = 14) Assigned male at birth (n = 5) Male (n = 1) Demiboy (n = 1) Female (n = 2) Transgender woman (n = 2) Nonbinary/Gender nonconforming/Genderqueer (n = 10) Genderqueer only (n = 3)	33 (range 22-59)	Semistructured interviews	Interpretive qualitative study Trans and queer theory Fat studies
Smith et al (2022)^80^	USA	n = 15 Transgender man (n = 7) Transgender woman (n = 4) Nonbinary (n = 4)	NR	Semistructured interviews	Consensual qualitative research
Sood et al (2021)^81^	USA	Transgender man (n = 2)	16 and 17	Case series	
Sun et al (2025)^82^	USA	n = 59 Nonbinary (n = 11) Transgender woman (n = 20) Transgender man (n = 24) Gender nonconforming (n = 1) Other (n = 3)	24-40	Survey	
Sun et al (2025)^83^	Online	604 posts and comments	NR	Reddit content analysis	Inductive thematic analysis
Tirrell et al (2023)^84^	USA	n = 103	27	Survey	
Tristani-Firouzi et al (2022)^85^	USA	n = 456 Transgender man (n = 298) Nonbinary (n = 158)	28 27	Anonymous survey	
Valente et al (2023)^86^	USA	n = 13 Transgender woman (n = 5) Women (n = 3) Man (n = 2) Nonbinary (n = 2) Genderqueer (n = 1)	31.3 ± 10.7 (range 18-52)	Semistructured interviews	Descriptive phenomenology
Valiquette (2024)^87^	Canada	n = 12 Transgender man (n = 1) Transgender woman (n = 5) Trans masculine (n = 1) Nonbinary (n = 2) Genderfluid (n = 1) Genderqueer (n = 1) Genderqueer trans masculine nonbinary (n = 1)	20-29 (n = 6) 30-39 (n = 3) 50+ (n = 1)	Observation and interview	Institutional ethnography
von Vogelsang et al (2016)^88^	Sweden	n = 6	NR	Semistructured interviews	Manifest qualitative content analysis
Weixel et al (2025)^89^	USA	n = 50 Transgender man (n = 45) Nonbinary (n = 4) Cisgender (n = 1)	20.0 ± 2.32 (range 16-25)	Chart review and survey	
Windt et al (2026)^90^	Australia	n = 9 Transgender man (n = 5) Transgender woman (n = 2) Nonbinary (n = 1) Cisgender woman (n = 1)	49.7 ± 11.5 (range 34-67)	Semistructured interviews	Exploratory
Yang et al (2025)^91^	Online	NR	NR	Reddit comments	Word cloud

**Table 2.** Critical Appraisal Skills Programme Qualitative Studies Checklist.

Study	Clear Statement of Research Aims?	Appropriate Qualitative Methodology?	Appropriate Research Design?	Appropriate Recruitment Strategy?	Appropriate Data Collection?	Adequate Consideration of Participant Researcher Relationship?	Adequate Consideration of Ethical Issues?	Data Analysis Sufficiently Rigorous?	Clear Statement of Findings?	Is the Research Valuable?
Raner et al(2024)^14^	yes	yes	yes	yes	yes	somewhat	yes	yes	yes	yes
Agarwal and Dhillon (2023)^33^	yes	yes	yes	yes	yes	yes	yes	yes	yes	yes
Bauer et al (2009)^34^	yes	yes	yes	somewhat	yes	somewhat	yes	yes	yes	yes
Bell and Purkey (2019)^35^	yes	yes	yes	yes	yes	somewhat	yes	yes	yes	yes
Bjørnson and Sagbakken (2023)^36^	yes	yes	yes	yes	yes	yes	yes	yes	yes	yes
Boyer et al (2023)^37^	yes	yes	yes	yes	yes	somewhat	yes	yes	yes	yes
Bridgwater et al (2024)^38^	yes	somewhat	somewhat	somewhat	somewhat	no	yes	somewhat	yes	yes
Cavic et al (2025)^39^	yes	yes	yes	yes	yes	yes	yes	yes	yes	yes
Chang et al (2023)^40^	yes	yes	yes	yes	yes	yes	yes	yes	yes	yes
Christiano et al (2024)^41^	yes	yes	yes	yes	yes	yes	yes	yes	yes	yes
Crabtree et al (2024)^42^	yes	yes	yes	yes	yes	yes	yes	yes	yes	yes
D'Angelo et al (2025)^43^	yes	yes	yes	yes	yes	somewhat	yes	yes	yes	yes
de Brouwer et al (2021)^44^	yes	yes	yes	yes	yes	yes	yes	yes	yes	yes
del Mar Pastor Bravo and Linander (2024)^45^	yes	yes	yes	yes	yes	somewhat	yes	yes	yes	yes
Dozier (2023)^46^	yes	yes	yes	yes	yes	yes	yes	yes	yes	yes
Dupasquier et al (2025)^47^	yes	yes	yes	yes	yes	somewhat	yes	yes	yes	yes
El-Hadi et al (2018)^48^	yes	yes	yes	yes	yes	yes	yes	yes	yes	yes
Ferrin et al (2024)^49^	yes	no	no	no	no	no	yes	no	yes	yes
Frohard-Dourlent et al (2020)^50^	yes	yes	yes	yes	yes	yes	yes	yes	yes	yes
Gillani et al (2025)^51^	yes	somewhat	somewhat	yes	somewhat	no	yes	somewhat	yes	yes
Goetz and Arcomano (2023)^52^	yes	yes	yes	yes	yes	somewhat	yes	yes	yes	yes
Harry-Hernandez et al (2020)^53^	yes	yes	yes	yes	yes	yes	yes	yes	yes	yes
Houghtaling (2023)^54^	yes	yes	yes	yes	yes	yes	yes	yes	yes	yes
Hu et al (2023)^55^	yes	somewhat	somewhat	somewhat	somewhat	no	yes	somewhat	yes	yes
Hu et al (2025)^56^	yes	yes	yes	yes	yes	somewhat	yes	yes	yes	yes
Katz-Wise et al (2017)^57^	yes	yes	yes	yes	yes	somewhat	yes	yes	yes	yes
Kloer et al (2023)^58^	yes	yes	yes	yes	yes	somewhat	yes	yes	yes	yes
Lee et al (2019)^59^	yes	yes	yes	yes	yes	yes	yes	yes	yes	yes
Lerri et al (2025)^60^	yes	yes	yes	yes	yes	somewhat	yes	yes	yes	yes
Littman (2021)^61^	somewhat	somewhat	no	no	somewhat	no	somewhat	no	somewhat	somewhat
MacCormick et al (2024)^62^	yes	yes	yes	yes	yes	yes	yes	yes	yes	yes
MacDonald et al (2016)^63^	yes	yes	yes	yes	yes	somewhat	yes	yes	yes	yes
Makhoul et al (2023)^64^	yes	yes	yes	yes	yes	yes	yes	yes	yes	yes
Martin and Coolhart (2022)^65^	yes	yes	yes	yes	yes	yes	yes	yes	yes	yes
Mathis (2021)^66^	yes	yes	yes	yes	yes	yes	yes	somewhat	yes	yes
McTernan et al (2020)^67^	yes	no	no	no	no	no	yes	no	yes	yes
Miller (2019)^68^	yes	yes	yes	yes	yes	yes	yes	somewhat	yes	yes
Mousavian et al (2024)^69^	yes	yes	yes	yes	yes	somewhat	yes	yes	yes	yes
Nieder et al (2021)^70^	yes	yes	yes	yes	yes	somewhat	yes	yes	yes	yes
Pipkin and Clarke (2024)^71^	yes	yes	yes	yes	yes	yes	yes	yes	yes	yes
Poudrier et al (2019)^72^	yes	no	no	yes	yes	somewhat	yes	somewhat	yes	yes
Puckett et al (2018)^73^	yes	somewhat	somewhat	somewhat	somewhat	yes	somewhat	somewhat	somewhat	yes
Raghuram (2024)^74^	yes	yes	yes	yes	yes	yes	yes	yes	yes	yes
Roller et al (2015)^75^	yes	yes	yes	yes	yes	somewhat	yes	yes	yes	yes
Ross et al (2024)^76^	yes	yes	yes	yes	yes	yes	yes	somewhat	yes	yes
Ross et al (2016)^77^	yes	no	no	yes	somewhat	no	yes	no	yes	yes
Schafer et al (2024)^78^	yes	yes	yes	yes	yes	somewhat	yes	yes	yes	yes
Slothouber (2024)^79^	yes	somewhat	somewhat	somewhat	somewhat	no	yes	somewhat	yes	yes
Smith et al (2022)^80^	yes	no	no	no	no	no	yes	no	yes	yes
Sood et al (2021)^81^	yes	no	no	no	no	no	yes	somewhat	yes	yes
Sun et al (2025)^82^	yes	somewhat	somewhat	yes	somewhat	no	yes	somewhat	yes	yes
Sun et al (2025)^83^	yes	no	no	yes	no	no	yes	no	yes	yes
Tirrell et al (2023)^84^	yes	yes	yes	yes	yes	somewhat	yes	yes	yes	yes
Tristani-Firouzi et al (2022)^85^	yes	somewhat	somewhat	somewhat	somewhat	no	yes	somewhat	yes	yes
Valente et al (2023)^86^	yes	yes	yes	yes	yes	yes	yes	somewhat	yes	yes
Valiquette (2024)^87^	yes	yes	yes	yes	yes	yes	yes	somewhat	yes	yes
von Vogelsang et al (2016)^88^	yes	yes	yes	yes	yes	yes	yes	yes	yes	yes
Weixel et al (2025)^89^	yes	somewhat	yes	yes	yes	somewhat	yes	somewhat	yes	yes
Windt et al (2026)^90^	yes	yes	yes	yes	yes	yes	yes	yes	yes	yes
Yang et al (2025)^91^	yes	yes	yes	yes	yes	yes	yes	yes	yes	yes

Note: This checklist provides a structured method of appraising the methodological rigor and validity of qualitative research. Adapted from the Critical Appraisal Skills Programme (CASP) Qualitative Checklist^27^ © CASP, licensed under CC BY-NC-SA 4.0 (https://creativecommons.org/licenses/by-nc-sa/4.0/). Formatting was modified and wording was changed to present findings in chart format. NR, not reported.

## Charting a Course

The analytical theme, charting a course, developed across three subthemes: confusion,^14,37,46,48,51,52,62,64,73,80^ financial strain,^14,37-39,43,46,48,52,62,64,68,73,79,80,84,85,87,90,91^ and community support.^33,37,39,43,45-47,51,62,66,68,71,73,75,77,84,86^ This theme appeared in 28 studies and was drawn from the experiences of at least 1358 patients as well as analysis of digital media (Table 3).

**Table 3.** Themes and Subthemes.

Theme	Subtheme	Participants (n)	Articles (n)	Articles
1. Charting a Course	Confusion	582	10	^14,37,46,48,51,52,62,64,73,80^
	Financial Strain	1190	19	^14,37-39,43,46,48,52,62,64,68,73,79,80,84,85,87,90,91^
	Community Support	607	17	^33,37,39,43,45-47,51,62,66,68,71,73,75,77,84,86^
	Total:	1358	28	^14,33,37-39,43,45-48,51,52,62,64,66,68,71,73,75,77,79,80,84-87,90,91^
2. Interacting with Providers	Negative Experiences	757	21	^14,34,35,37,38,40,43,45-47,51,52,54,59,62,64,69,77,80,81,87^
	Gatekeepers	1361	26	^14,35,38,45-48,50-52,54,55,57,62,63,71,73-75,80,84,85,87,88,90,91^
	Performing Transness	1273	20	^14,35,45,47,50-52,54,55,57,62,63,73,75,80,84,85,87,90,91^
	Protective Factors	219	12	^38,41,47,50,59,62,65,69,77,80,86,88^
	Total:	1835	38	^14,34,35,37,38,40,41,43,45-48,50-52,54,55,57,59,62-65,69,71,73-75,77,80,81,84-88,90,91^
3. Surgical goal setting		550	14	^14,38,47-50,52,55,72,75,79,82,87,91^
4. Adjusting to Oneself	Relief	669	15	^33,36,39,41,42,44,48,52,56,65,68,72,76,81,83^
	Unmet Expectations	593	8	^42,44,52,56,66,70,76,83^
	Lifelong Process	113	2	^52,82^
	Total	825	18	^33,36,39,41,42,44,48,52,56,65,66,68,70,72,76,81-83^

### Confusion

Study participants consistently reported uncertainty about where to start, how to locate surgeons, and how to navigate the insurance and referral processes when accessing gender-affirming care.^14,37,46,48,51,52,62,64,73,80^ “It wasn’t that the steps were overly difficult. Half the battle was figuring out what the steps were.”^62^ Other challenges included unclear timelines for surgery approvals, denials, and short validity periods for funding, where something as small as missing a single form could delay surgery indefinitely.^69^ Socioeconomic factors shaped the ability of patients to manage these barriers.^56^

### Financial

Across studies, cost frequently arose as one of the most significant and persistent barriers to access.^14,37-39,43,46,48,52,62,64,68,73,79,80,84,85,87,90,91^ Indirect expenses such as parking and unpaid recovery time off work created substantial challenges. These were compounded for patients in rural settings, with some participants reported traveling hours for their appointments.^73^ Participants described concerns related to two-tier healthcare systems, in which publicly funded care was perceived as inferior to private options.^68^

### Community

Connections with people who had already transitioned helped participants navigate their own processes. Peer networks, online forums, and social media groups were frequently described as primary sources of information and support.^33,37,45^ Providers were identified using word-of-mouth recommendations, with positive and negative reputations circulating through informal networks. “It was like hunting down information and asking people different questions about their experiences with different surgeons and different procedures.”^51^

Informal and formal navigators such as parents, friends, and specialized LGBTQ+ health centers provided important assistance. Patients relied on professional contacts with relevant expertise to appeal denials or secure coverage, for example.^46^ Patients consistently reported smoother access to care when working within their support networks.^33,37,39,43,45,62,86^

## Interactions with Providers

The theme of Interactions with Providers was the most prevalent theme identified in the present review. This analytical theme developed across four subthemes: negative experiences,^4,34,35,37,38,40,43,45-47,51,52,54,59,62,64,69,77,80,81,87^ gatekeepers,^14,35,38,45-48,50-52,54,55,57,62,63,71,73-75,80,84,85,87,88,90,91^ performing transness,^14,35,45,47,50-52,54,55,57,62,63,73,75,80,84,85,87,90,91^ and protective factors.^38,41,47,50,59,62,65,69,77,80,86,88^ It appeared across 38 studies and drew on the experiences of at least 1835 participants as well as digital media (Table 3).

### Negative Experiences

Across studies, healthcare encounters were marked by limited provider knowledge and avoidance when addressing trans-specific health concerns. This left many patients feeling dismissed or placed in the uncomfortable position of having to explain basic aspects of transgender care to their providers. Patients were often redirected to specialists because physicians were “not comfortable working with trans patients,” noting that even when information was offered, some providers “had no interest to learn.”^34^ Being repeatedly “passed along” between providers disrupted continuity of care and, in many cases, created the ongoing fear that physicians would refuse to provide necessary care.^34,38,54^

Negative encounters ranged from invasive questioning to perceived or anticipated discrimination.^40,62^ Misgendering contributed to feelings of invisibility, frustration, and mistrust, particularly for racialized patients, and patients who were early in their transition.

Experiences of trauma in healthcare settings shaped subsequent engagement with healthcare providers. Participants reported anticipating transphobia during routine appointments, which led them to avoid seeking medical care.^38,42,55^ The cumulative effect of this trauma was heightened vigilance and stress during healthcare interactions.

### Gatekeepers

This theme illustrates the perception of healthcare providers as gatekeepers. Patients described a “power imbalance to deny or not deny.”^91^ This was articulated as a loss of agency; transgender individuals did not feel capable of asserting themselves in assessments because they were afraid of being denied care.^50^ Patients contrasted the lack of agency in these assessments with the autonomy afforded to cisgender individuals undergoing elective procedures, noting that transgender people are often treated as needing protection from their own decisions.^50^

This fear led some patients to tell assessors what they wanted to hear, including altering information about themselves, such as sexual orientation and mental health, to avoid being deemed ineligible.^47,90^ This was based on the belief that many providers based approval for gender-affirming surgery on their own biased judgement, as opposed to following guidelines

### Performing Transness

Study participants often described the need to perform a stereotypical or textbook version of transness in order to satisfy assessors. This was an active performance; participants practiced “fake interviews” with peers to ensure they said the “right things.”^35,48,62^ This experience was widely described as alienating and emotionally draining.

Nonbinary patients frequently reported feeling pressured to conform to binary narratives in order to access surgery.^51^ Many hid their identity to meet assessors’ expectations: “I had an expectation to play the system; resulted in me coming out as a trans male, despite that wasn’t how I identified so that I could get the surgery I needed.”^47^ Nonbinary participants also critiqued the rigid expectation to present a “textbook” transgender narrative, which often forced them to compromise on their care goals.^54^ “Many healthcare providers I encountered seem to have a view that trans people are ‘switching genders’ and still work within a binary system of male/female. I was very scared that I would not present as ‘male enough’ to qualify for surgery and funding.”^14^

### Protective Factors

Within this broader pattern of deficit, patients also identified characteristics of positive interactions with healthcare providers. Healthcare providers’ knowledge and attitudes fundamentally shaped transgender patients’ capacity to move through healthcare systems.^86^ Physicians who were “open-minded and happy to learn things to fill in their gaps” or who possessed strong medical knowledge coupled with cultural awareness were viewed as facilitating affirming and navigable care experiences.^59,86^ Similarly, positive surgical readiness assessments felt like nonconfrontational conversations, with positive surgical assessors described as communicative, accepting, and knowledgeable. Healthcare provider competence was observed to foster trust and enable smoother navigation of the care pathway.

## Surgical Goal Setting

The analytical theme, Surgical Goal Setting, appeared in only 14 studies and drew from the experiences of 550 participants, in addition to digital media and forum posts.^14,38,47-50,52,55,72,75,79,82,87,91^ Surgical Goal Setting drew solely on studies from Canada and the USA (Table 3).

There were distinct differences in surgical goals between binary transgender men and gender-diverse study participants. Binary transgender men predominantly pursued chest masculinization surgery with the goal of achieving a traditionally masculine chest, as one participant explained, “I wanted a chest to look like a normal cis male … Specifically for me that included smaller nipples and areolas.”^14^

In contrast, gender diverse (or nonbinary) individuals demonstrated more variability in their surgical goals. While some wanted a flat chest, others aimed for an androgynous or flexible appearance that did not conform to traditional masculine or feminine ideals. Surgical goals were often driven by a desire to reduce dysphoria and to maintain a body congruent with a nonbinary identity rather than to conform to binary norms.^52^ Some nonbinary individuals elected to partially conserve breast mass in order to present flexibly.^14^ Nonbinary participants’ approaches to nipple sensation were also highly individualized. Some nonbinary participants valued the preservation of nipple sensation, while others opted for no nipples entirely.^52,91^ In contrast, binary transgender men often regarded the preservation or loss of nipple sensation as secondary to attaining the desired flat, masculine appearance.

Some patients were hesitant to pursue surgery due to the anticipated challenges of meeting BMI cutoffs.^79,82,90^ Reduction mammaplasty was sometimes considered as an alternative or interim procedure to full chest masculinization surgery, particularly when social or logistical barriers limited access.^38,47,75^

## Adjusting to Oneself

This analytical theme captured how individuals responded to the aftermath of chest masculinization, and developed across three subthemes: relief,^33,36,39,41,42,44,48,52,56,65,68,72,76,81,83^ unmet expectations,^42,44,52,56,66,70,76,83^ and lifelong process.^52,82^ This theme appeared in 18 studies and drew on the experiences of 825 participants as well as digital media and forum posts (Table 3).

### Relief

Study participants described the profound relief of no longer experiencing longstanding gender dysphoria.^42,48^ Beyond alleviating dysphoria, participants also described chest masculinization surgery as a turning point with, “all these things are opening up for me.”^76^

### Unmet Expectations

Despite largely positive experiences, a small number of patients reported unmet expectations. Dissatisfaction often stemmed from esthetic outcomes or lack of postoperative support.^44^ Participants whose surgical outcomes were disappointing reported continued bodily incongruence and dysphoria. Participants also described feeling lost when coordinating their follow-up appointments.

### Lifelong Process

Finally, postoperative experiences were overwhelmingly positive, consistent with quantitative research demonstrating the benefits of gender-affirming surgery.^6^ For many, surgery was not viewed as an endpoint but as part of an ongoing process of identity integration.^52^ While some participants worried about surgical scars being noticeable markers, others expressed pride in their recovery and in visible signs of surgical transformation. Several patients also described a deepened sense of belonging within transgender communities. This belonging is outlined by one participant: “I’ll always be trans, right? There's no end point to that … my body's never going to be a normative one. And that's cool. That's beautiful.”^52^

## Discussion

**Figure 2.**
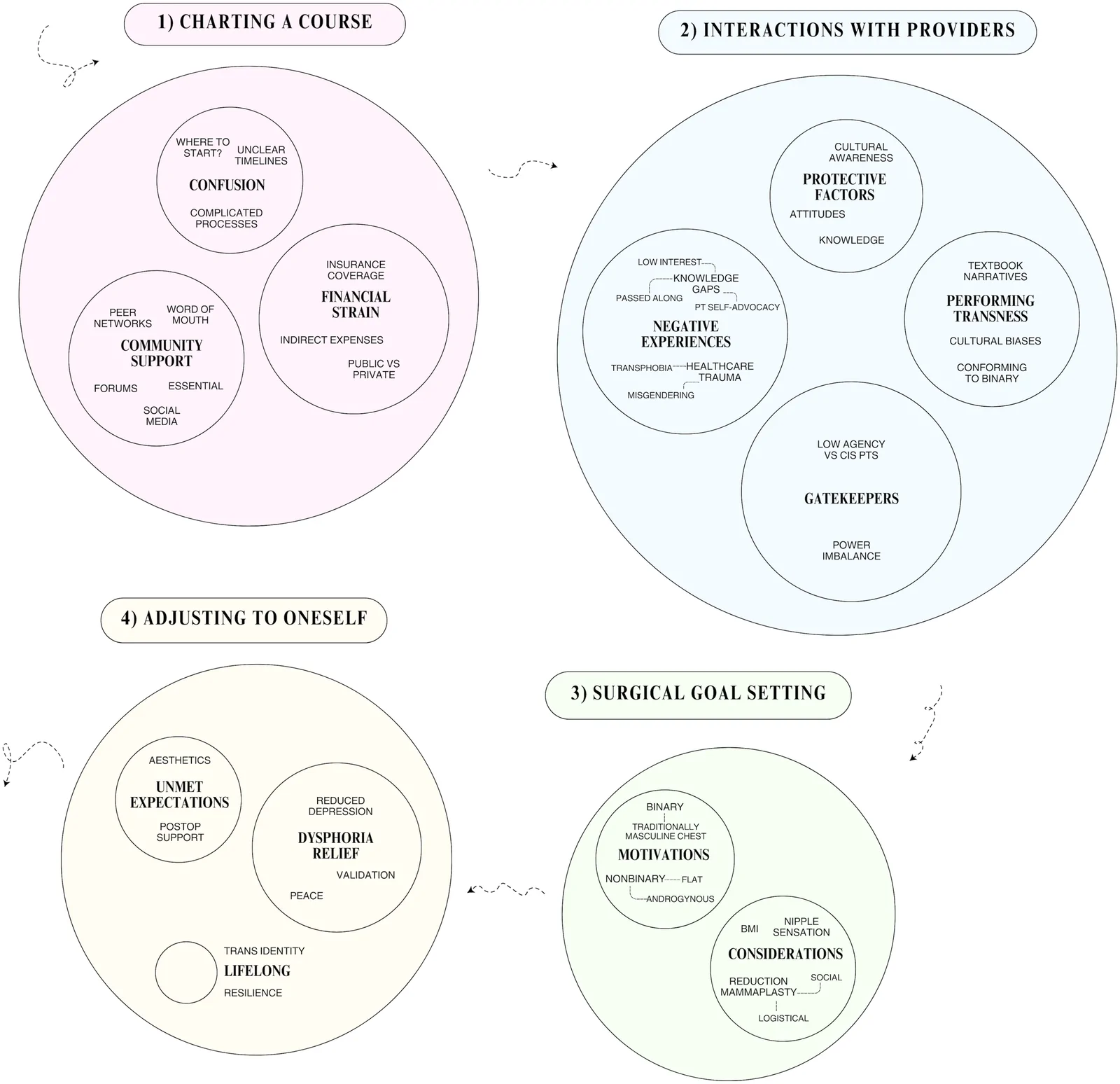
Conceptual relationships among analytical themes shaping access to gender-affirming chest masculinization surgery. The area of each circle is proportional to the number of studies cited for each theme and subtheme. BMI, body mass index.

This systematic review and thematic synthesis explored the lived experiences of TGD individuals throughout the process of undergoing chest masculinization surgery (Figure 2). The experiences described in this review are intrinsically linked to marginalization. Intersectionality, a theoretical framework developed by Crenshaw in the 1980s,^92^ provides a lens through which we interpret the compounding nature of the disadvantages experienced by TGD people as they navigate access to gender-affirming care.^93,94^ Similarly, minority stress theory posits that the chronic exposure to discrimination increases stress, leading to health disparities among marginalized populations.^95-98^ This was demonstrated in our findings, where the widespread lack of formal training for providers may compound the stress of seeking gender-affirming care, with each repeated negative experience priming an individual to be more harmed by a single interaction.^99,100^

Despite research showing that gender-affirming procedures improve quality of life, reduce gender incongruence, and are cost-effective, these procedures are subject to extensive debate and insurance denial rates remain high, which particularly affected participants from the USA.^101,102^ For context, the median costs for gender-affirming chest-surgery were $29,887 USD, according to a nationwide American survey by Das et al with 90.7% (n = 19,308) covered at least in part by private or public health insurance.^103-104^ In Canada, masculinizing chest surgery is currently covered nationwide, feminizing chest surgery is covered under limited conditions, while procedures like chest-contouring liposuction or reduction are rarely covered by government insurance.^79,104,105^ Chest masculinizing procedures are sometimes classified as “reconstructive” or “esthetic,” further affecting coverage. The theme of having to chart a course to access care was evident in the costs incurred.

A critical finding of our synthesis is that transgender and nonbinary patients represent populations with often distinct surgical priorities. While transgender men generally sought a traditionally masculine appearance, the goals of gender diverse participants spanned a much wider spectrum. These observations were substantiated by Schafer et al., who found that nonbinary patients frequently request preservation of nipple-areola complexes, small breast mounds, or other androgynous features (such as removal of nipples entirely).^78^

When seeking gender-affirming care, the burden of navigation is in and of itself a psychosocial stressor. Previous research has identified the lack of knowledgeable and willing providers as the most pervasive barrier to gender-affirming care, and the scarcity of trained providers results in patients approaching medical encounters with heightened vigilance and mistrust.^106,107^ This deficit has been recognized by physicians themselves, with many acknowledging a structural failure of medical curricula to equip them with the ability to manage transgender health.^108^

Similarly, Mikulak et al record the accounts of providers which closely mirror the descriptive theme of negative experiences developed from accounts of patients, where providers report inadequate training, long waiting lists, and the absence of clear clinical guidelines as central barriers to their ability to provide care.^109^ These deficits provide an explanation for the interpersonal strain observed in our synthesis.

However, interactions with healthcare providers during the process of obtaining gender-affirming care are fundamentally distinct from interactions in other healthcare contexts. The perception of providers as gatekeepers creates a kind of anticipatory anxiety, where patients feel pressure to present a version of their gender identity that would be deemed acceptable to clinicians or funding bodies. They often concealed the presence or absence of mental health concerns or their sexual orientation to avoid being denied surgery. We note that this expectation to “perform” transness places exceptional emotional strain on nonbinary individuals whose identities do not fit established transformative frameworks.

Training healthcare professionals on transgender health was identified as an area likely to be highly impactful across multiple contexts.^110^ Patients across studies stressed that the responsibility for education should not have to fall to transgender patients.^50^ We note that affirming provider interactions had a significant positive impact. When providers approached care with openness and competence, participants felt validated, informed, and empowered. Improving access therefore requires structural and educational reforms.

Based on our findings, we recommend integrating transgender health education across medical training and continuing professional development, specifically with regards to interacting with transgender patients and with respect to the care pathway. As well, we recommend that surgical planning explicitly outline each patient's individual goals, rather than assuming a uniform or binary ideal chest outcome, in order to better support patient-centric care.

## Limitations

Most included studies were conducted in high-income Western countries, limiting applicability to other contexts. CASP appraisal found several methodological limitations such as a lack of detail regarding recruitment strategies.^27^ Many studies (n = 14 not at all, n = 20 somewhat) did not detail author positionality, which may further cisnormative assumptions. In addition, our study focuses on patient experiences and does not assess the experiences of healthcare providers themselves. Healthcare practitioners who provide gender-affirming care face substantial challenges, including lack of institutional resources, administrative and legal pressures, evolving standards of care, and threats to personal or professional safety.^111^ These challenges may contribute to care environments where patient needs and provider well-being are at odds. Our current study assesses just one of the populations impacted by barriers to care. Future work assessing the perspectives of care providers is warranted to fully access to and delivery of gender-affirming care.

## Conclusion

The experiences of TGD individuals accessing masculinizing chest surgery are determined by structural barriers, the interpersonal dynamics between patients and providers, and also by the diverse goals and identities of TGD individuals. Informed providers help TGD patients navigate the system; they are able to chart a course for their patients. The four analytical themes which emerged through our analysis identify persistent structural barriers to care but also highlight the resilience and resourcefulness of TGD patients.

## Supplemental Material

sj-docx-1-psg-10.1177_22925503261477385 - Supplemental material for Experiences of Transgender and Gender Diverse Patients in Accessing Gender-Affirming Chest Masculinization: A Systematic Review and Qualitative Thematic Synthesis of the Literature Supplemental material, sj-docx-1-psg-10.1177_22925503261477385 for Experiences of Transgender and Gender Diverse Patients in Accessing Gender-Affirming Chest Masculinization: A Systematic Review and Qualitative Thematic Synthesis of the Literature by Emma Lim, Justin Haas, Precious Adekoya, Sophia Hou, Kathleen Armstrong, Helene Retrouvey and Ammara Ghumman in Plastic Surgery
