# Supplementary material for: Experiences of Transgender and Gender Diverse Patients in Accessing Gender-Affirming Chest Masculinization: A Systematic Review and Qualitative Thematic Synthesis of the Literature
Source: Plast Surg (Oakv). 2026 Aug 26:22925503261477385. Online ahead of print. doi: 10.1177/22925503261477385 (PMC13518616; doi:10.1177/22925503261477385)
Supplement: Supplementary file 1 [file sj-docx-1-psg-10.1177_22925503261477385.docx]

**Supplementary Data**

| MEDLINE | ("transgender man*" OR transman* OR "female-to-male" OR FTM OR nonbinary OR "non-binary" OR "gender nonconforming" OR "gender diverse" OR genderqueer OR "trans masculine" OR transmasculine OR gender identit* OR gender nonconform* OR gender express* OR gender atypical OR gender bend* OR gender creative* OR gender independen* OR intersex* OR gender fluid* OR gender flex* OR transexual*)  AND  ("top surgery" OR "gender affirming surgery" OR "gender-affirming surgery" OR "gender confirmation surgery" OR mastectomy OR "subcutaneous mastectomy" OR "simple mastectomy" OR "double incision mastectomy" OR "keyhole mastectomy" OR "peri-areolar mastectomy" OR "chest reconstruction" OR "chest masculinization" OR "chest contouring" OR "free nipple graft" OR "nipple graft" OR "nipple reconstruction" OR "gender affirming mastectomy" OR "transgender mastectomy")  AND  ("healthcare interaction*" OR "healthcare utilization" OR "healthcare experienc*" OR "healthcare access" OR "experience*" OR "health service*" OR "patient preferenc* OR "Patient* experience*" OR "patient* perception*" OR "patient* perspective " OR "barrier*" OR "facilitator*" OR "quality of life" OR "patient satisfaction" OR "patient engagement" OR "care experience*") |
| --- | --- |
| EMBASE | exp transgender person/ or exp transsexualism/ or (intersex$ or transgender man$ or transman$ or female-to-male or FTM or nonbinary or non-binary or gender nonconforming or gender diverse or genderqueer or trans masculine or transmasculine or gender identit$ or gender nonconform$ or gender express$ or gender atypical or gender bend$ or gender creative$ or gender independen$ or gender fluid$ or gender flex$ or transexual$).ti,ab.  AND  exp gender reassignment surgery/ or exp mastectomy/ or (top surgery or gender affirming surgery or gender-affirming surgery or gender confirmation surgery or mastectomy or subcutaneous mastectomy or simple mastectomy or double incision mastectomy or keyhole mastectomy or peri-areolar mastectomy or chest reconstruction or chest masculinization or chest contouring or free nipple graft or nipple graft or nipple reconstruction or gender affirming mastectomy or transgender mastectomy).ti,ab.  AND  exp health care utilization/ or exp patient experience/ or exp quality of life/ or exp health care access/ or exp patient satisfaction/ or exp patient engagement/ or (healthcare interaction$ or healthcare utilization or healthcare experienc$ or healthcare access or experience$ or health service$ or patient preferenc$ or patient$ experience$ or patient$ perception$ or patient$ perspective or barrier$ or facilitator$ or quality of life or patient satisfaction or patient engagement or care experience$).ti,ab. |
| SCOPUS | (  TITLE-ABS-KEY("transgender man*" OR transman* OR "female-to-male" OR FTM OR nonbinary OR "non-binary" OR "gender nonconforming" OR "gender diverse" OR genderqueer OR "trans masculine" OR transmasculine OR gender identit* OR gender nonconform* OR gender express* OR gender atypical OR gender bend* OR gender creative* OR gender independen* OR intersex* OR gender fluid* OR gender flex* OR transexual*)  )  AND  (  TITLE-ABS-KEY("top surgery" OR "gender affirming surgery" OR "gender-affirming surgery" OR "gender confirmation surgery" OR mastectomy OR "subcutaneous mastectomy" OR "simple mastectomy" OR "double incision mastectomy" OR "keyhole mastectomy" OR "peri-areolar mastectomy" OR "chest reconstruction" OR "chest masculinization" OR "chest contouring" OR "free nipple graft" OR "nipple graft" OR "nipple reconstruction" OR "gender affirming mastectomy" OR "transgender mastectomy")  )  AND  (  TITLE-ABS-KEY("healthcare interaction*" OR "healthcare utilization" OR "healthcare experienc*" OR "healthcare access" OR experience* OR "health service*" OR "patient preferenc*" OR "patient* experience*" OR "patient* perception*" OR "patient* perspective" OR barrier* OR facilitator* OR "quality of life" OR "patient satisfaction" OR "patient engagement" OR "care experience*") |
| WebofScience | TS=(  "transgender man*" OR transman* OR "female-to-male" OR FTM OR nonbinary OR "non-binary" OR "gender nonconforming" OR "gender diverse" OR genderqueer OR "trans masculine" OR transmasculine OR gender identit* OR gender nonconform* OR gender express* OR gender atypical OR gender bend* OR gender creative* OR gender independen* OR intersex* OR gender fluid* OR gender flex* OR transexual*  )  AND TS=(  "top surgery" OR "gender affirming surgery" OR "gender-affirming surgery" OR "gender confirmation surgery" OR mastectomy OR "subcutaneous mastectomy" OR "simple mastectomy" OR "double incision mastectomy" OR "keyhole mastectomy" OR "peri-areolar mastectomy" OR "chest reconstruction" OR "chest masculinization" OR "chest contouring" OR "free nipple graft" OR "nipple graft" OR "nipple reconstruction" OR "gender affirming mastectomy" OR "transgender mastectomy"  )  AND TS=(  "healthcare interaction*" OR "healthcare utilization" OR "healthcare experienc*" OR "healthcare access" OR experience* OR "health service*" OR "patient preferenc*" OR "patient* experience*" OR "patient* perception*" OR "patient* perspective" OR barrier* OR facilitator* OR "quality of life" OR "patient satisfaction" OR "patient engagement" OR "care experience*"  ) |
| CINAHL | (MH "Transgender Persons" OR MH "Transsexualism" OR MH "Gender Identity" OR MH "Gender Dysphoria" OR TI "transgender man*" OR AB "transgender man*" OR TI transman* OR AB transman* OR TI "female-to-male" OR AB "female-to-male" OR TI FTM OR AB FTM OR TI nonbinary OR AB nonbinary OR TI "non-binary" OR AB "non-binary" OR TI "gender nonconforming" OR AB "gender nonconforming" OR TI "gender diverse" OR AB "gender diverse" OR TI genderqueer OR AB genderqueer OR TI "trans masculine" OR AB "trans masculine" OR TI transmasculine OR AB transmasculine OR TI "gender identit*" OR AB "gender identit*" OR TI "gender nonconform*" OR AB "gender nonconform*" OR TI "gender express*" OR AB "gender express*" OR TI "gender atypical" OR AB "gender atypical" OR TI "gender bend*" OR AB "gender bend*" OR TI "gender creative*" OR AB "gender creative*" OR TI "gender independen*" OR AB "gender independen*" OR TI intersex* OR AB intersex* OR TI "gender fluid*" OR AB "gender fluid*" OR TI "gender flex*" OR AB "gender flex*" OR TI transexual* OR AB transexual*)  (MH "Mastectomy" OR MH "Gender Affirming Surgery" OR TI "top surgery" OR AB "top surgery" OR TI "gender affirming surgery" OR AB "gender affirming surgery" OR TI "gender-affirming surgery" OR AB "gender-affirming surgery" OR TI "gender confirmation surgery" OR AB "gender confirmation surgery" OR TI mastectomy OR AB mastectomy OR TI "subcutaneous mastectomy" OR AB "subcutaneous mastectomy" OR TI "simple mastectomy" OR AB "simple mastectomy" OR TI "double incision mastectomy" OR AB "double incision mastectomy" OR TI "keyhole mastectomy" OR AB "keyhole mastectomy" OR TI "peri-areolar mastectomy" OR AB "peri-areolar mastectomy" OR TI "chest reconstruction" OR AB "chest reconstruction" OR TI "chest masculinization" OR AB "chest masculinization" OR TI "chest contouring" OR AB "chest contouring" OR TI "free nipple graft" OR AB "free nipple graft" OR TI "nipple graft" OR AB "nipple graft" OR TI "nipple reconstruction" OR AB "nipple reconstruction" OR TI "gender affirming mastectomy" OR AB "gender affirming mastectomy" OR TI "transgender mastectomy" OR AB "transgender mastectomy")  (MH "Health Services Accessibility" OR MH "Health Services Utilization" OR MH "Patient Satisfaction" OR MH "Patient Experience" OR MH "Quality of Life" OR MH "Barriers to Care" OR MH "Facilitators of Health Care" OR TI "healthcare interaction*" OR AB "healthcare interaction*" OR TI "healthcare utilization" OR AB "healthcare utilization" OR TI "healthcare experienc*" OR AB "healthcare experienc*" OR TI "healthcare access" OR AB "healthcare access" OR TI experience* OR AB experience* OR TI "health service*" OR AB "health service*" OR TI "patient preferenc*" OR AB "patient preferenc*" OR TI "patient* experience*" OR AB "patient* experience*" OR TI "patient* perception*" OR AB "patient* perception*" OR TI "patient* perspective" OR AB "patient* perspective" OR TI barrier* OR AB barrier* OR TI facilitator* OR AB facilitator* OR TI "quality of life" OR AB "quality of life" OR TI "patient satisfaction" OR AB "patient satisfaction" OR TI "patient engagement" OR AB "patient engagement" OR TI "care experience*" OR AB "care experience*")  S1 AND S2 AND S3 |
| PsychINFO | (DE "Transgender Persons" OR DE "Transsexualism" OR DE "Gender Identity" OR DE "Gender Dysphoria" OR TI "transgender man*" OR AB "transgender man*" OR TI transman* OR AB transman* OR TI "female-to-male" OR AB "female-to-male" OR TI FTM OR AB FTM OR TI nonbinary OR AB nonbinary OR TI "non-binary" OR AB "non-binary" OR TI "gender nonconforming" OR AB "gender nonconforming" OR TI "gender diverse" OR AB "gender diverse" OR TI genderqueer OR AB genderqueer OR TI "trans masculine" OR AB "trans masculine" OR TI transmasculine OR AB transmasculine OR TI "gender identit*" OR AB "gender identit*" OR TI "gender nonconform*" OR AB "gender nonconform*" OR TI "gender express*" OR AB "gender express*" OR TI "gender atypical" OR AB "gender atypical" OR TI "gender bend*" OR AB "gender bend*" OR TI "gender creative*" OR AB "gender creative*" OR TI "gender independen*" OR AB "gender independen*" OR TI intersex* OR AB intersex* OR TI "gender fluid*" OR AB "gender fluid*" OR TI "gender flex*" OR AB "gender flex*" OR TI transexual* OR AB transexual*)  (DE "Sex Reassignment Surgery" OR DE "Mastectomy" OR TI "top surgery" OR AB "top surgery" OR TI "gender affirming surgery" OR AB "gender affirming surgery" OR TI "gender-affirming surgery" OR AB "gender-affirming surgery" OR TI "gender confirmation surgery" OR AB "gender confirmation surgery" OR TI mastectomy OR AB mastectomy OR TI "subcutaneous mastectomy" OR AB "subcutaneous mastectomy" OR TI "simple mastectomy" OR AB "simple mastectomy" OR TI "double incision mastectomy" OR AB "double incision mastectomy" OR TI "keyhole mastectomy" OR AB "keyhole mastectomy" OR TI "peri-areolar mastectomy" OR AB "peri-areolar mastectomy" OR TI "chest reconstruction" OR AB "chest reconstruction" OR TI "chest masculinization" OR AB "chest masculinization" OR TI "chest contouring" OR AB "chest contouring" OR TI "free nipple graft" OR AB "free nipple graft" OR TI "nipple graft" OR AB "nipple graft" OR TI "nipple reconstruction" OR AB "nipple reconstruction" OR TI "gender affirming mastectomy" OR AB "gender affirming mastectomy" OR TI "transgender mastectomy" OR AB "transgender mastectomy")  (DE "Health Care Utilization" OR DE "Access to Health Care" OR DE "Health Care Delivery" OR DE "Patient Satisfaction" OR DE "Quality of Life" OR DE "Health Services Accessibility" OR DE "Barriers" OR DE "Facilitators" OR TI "healthcare interaction*" OR AB "healthcare interaction*" OR TI "healthcare utilization" OR AB "healthcare utilization" OR TI "healthcare experienc*" OR AB "healthcare experienc*" OR TI "healthcare access" OR AB "healthcare access" OR TI experience* OR AB experience* OR TI "health service*" OR AB "health service*" OR TI "patient preferenc*" OR AB "patient preferenc*" OR TI "patient* experience*" OR AB "patient* experience*" OR TI "patient* perception*" OR AB "patient* perception*" OR TI "patient* perspective" OR AB "patient* perspective" OR TI barrier* OR AB barrier* OR TI facilitator* OR AB facilitator* OR TI "quality of life" OR AB "quality of life" OR TI "patient satisfaction" OR AB "patient satisfaction" OR TI "patient engagement" OR AB "patient engagement" OR TI "care experience*" OR AB "care experience*") |
| SSCI | TS=(  "transgender man*" OR transman* OR "female-to-male" OR FTM OR nonbinary OR "non-binary" OR "gender nonconforming" OR "gender diverse" OR genderqueer OR "trans masculine" OR transmasculine OR gender identit* OR gender nonconform* OR gender express* OR gender atypical OR gender bend* OR gender creative* OR gender independen* OR intersex* OR gender fluid* OR gender flex* OR transexual*  )  AND TS=(  "top surgery" OR "gender affirming surgery" OR "gender-affirming surgery" OR "gender confirmation surgery" OR mastectomy OR "subcutaneous mastectomy" OR "simple mastectomy" OR "double incision mastectomy" OR "keyhole mastectomy" OR "peri-areolar mastectomy" OR "chest reconstruction" OR "chest masculinization" OR "chest contouring" OR "free nipple graft" OR "nipple graft" OR "nipple reconstruction" OR "gender affirming mastectomy" OR "transgender mastectomy"  )  AND TS=(  "healthcare interaction*" OR "healthcare utilization" OR "healthcare experienc*" OR "healthcare access" OR experience* OR "health service*" OR "patient preferenc*" OR "patient* experience*" OR "patient* perception*" OR "patient* perspective" OR barrier* OR facilitator* OR "quality of life" OR "patient satisfaction" OR "patient engagement" OR "care experience*"  ) |
| LGBTQ+ Source (EBSCOhost) | (SU "Transgender People" OR SU "Transmasculine People" OR SU "Gender Identity" OR SU "Nonbinary People" OR SU "Intersex People" OR TI "transgender man*" OR AB "transgender man*" OR TI transman* OR AB transman* OR TI "female-to-male" OR AB "female-to-male" OR TI FTM OR AB FTM OR TI nonbinary OR AB nonbinary OR TI "non-binary" OR AB "non-binary" OR TI "gender nonconforming" OR AB "gender nonconforming" OR TI "gender diverse" OR AB "gender diverse" OR TI genderqueer OR AB genderqueer OR TI "trans masculine" OR AB "trans masculine" OR TI transmasculine OR AB transmasculine OR TI "gender identit*" OR AB "gender identit*" OR TI "gender nonconform*" OR AB "gender nonconform*" OR TI "gender express*" OR AB "gender express*" OR TI "gender atypical" OR AB "gender atypical" OR TI "gender bend*" OR AB "gender bend*" OR TI "gender creative*" OR AB "gender creative*" OR TI "gender independen*" OR AB "gender independen*" OR TI intersex* OR AB intersex* OR TI "gender fluid*" OR AB "gender fluid*" OR TI "gender flex*" OR AB "gender flex*" OR TI transexual* OR AB transexual*)  (TI "top surgery" OR AB "top surgery" OR TI "gender affirming surgery" OR AB "gender affirming surgery" OR TI "gender-affirming surgery" OR AB "gender-affirming surgery" OR TI "gender confirmation surgery" OR AB "gender confirmation surgery" OR TI mastectomy OR AB mastectomy OR TI "subcutaneous mastectomy" OR AB "subcutaneous mastectomy" OR TI "simple mastectomy" OR AB "simple mastectomy" OR TI "double incision mastectomy" OR AB "double incision mastectomy" OR TI "keyhole mastectomy" OR AB "keyhole mastectomy" OR TI "peri-areolar mastectomy" OR AB "peri-areolar mastectomy" OR TI "chest reconstruction" OR AB "chest reconstruction" OR TI "chest masculinization" OR AB "chest masculinization" OR TI "chest contouring" OR AB "chest contouring" OR TI "free nipple graft" OR AB "free nipple graft" OR TI "nipple graft" OR AB "nipple graft" OR TI "nipple reconstruction" OR AB "nipple reconstruction" OR TI "gender affirming mastectomy" OR AB "gender affirming mastectomy" OR TI "transgender mastectomy" OR AB "transgender mastectomy")  (SU "Health Care Access" OR SU "Health Care Utilization" OR SU "Patient Satisfaction" OR SU "Quality of Life" OR SU "Patient Experience" OR TI "healthcare interaction*" OR AB "healthcare interaction*" OR TI "healthcare utilization" OR AB "healthcare utilization" OR TI "healthcare experienc*" OR AB "healthcare experienc*" OR TI "healthcare access" OR AB "healthcare access" OR TI experience* OR AB experience* OR TI "health service*" OR AB "health service*" OR TI "patient preferenc*" OR AB "patient preferenc*" OR TI "patient* experience*" OR AB "patient* experience*" OR TI "patient* perception*" OR AB "patient* perception*" OR TI "patient* perspective" OR AB "patient* perspective" OR TI barrier* OR AB barrier* OR TI facilitator* OR AB facilitator* OR TI "quality of life" OR AB "quality of life" OR TI "patient satisfaction" OR AB "patient satisfaction" OR TI "patient engagement" OR AB "patient engagement" OR TI "care experience*" OR AB "care experience*") |
